# Molecular Insights into the Mechanism of Caffeic Acid in Meat Color Improvement via Multispectral Spectroscopy and Molecular Dynamics Simulation

**DOI:** 10.3390/foods15193365

**Published:** 2026-09-22

**Authors:** Jianzeng Xin, Yiming Wang, Xiangku Ye, Sheng Liu

**Affiliations:** 1 School of Life Science, Yantai University, Yantai 264006, China; xinjianzeng@ytu.edu.cn (J.X.); yexk@163.com (X.Y.); 2 School of Food and Bioengineering, Weifang Vocational College of Food Science and Technology, Weifang 262100, China; wymytu@126.com; 3 School of Pharmacy, Yantai University, Yantai 264005, China

**Keywords:** meat color stability, Myoglobin, caffeic acid (CA), interaction, multi-spectroscopy, molecular dynamics simulation

## Abstract

This study investigated the color-stabilizing effects of caffeic acid (CA) on meat and its interaction mechanism with myoglobin (Mb) using in situ and in vitro model assays, combined with physicochemical analyses, multi-spectroscopic techniques, molecular docking, and molecular dynamics (MD) simulations. Three 24-month-old Simmental crossbred steer carcasses were utilized, yielding 12 steaks (four per carcass). The steaks were divided into three treatment groups: a control (deionized water), 0.05% (*w*/*v*) CA, and 0.1% (*w*/*v*) CA. Samples were stored at 4 ± 0.5 °C for 7 days under simulated retail display conditions, overwrapped with polyvinyl chloride (PVC) film. Storage assays demonstrated that 0.1% CA significantly delayed beef browning and maintained its red color. Physicochemical analyses revealed that CA inhibited metmyoglobin (MetMb) formation by 6%, reduced Mb solubility, and decreased surface hydrophobicity by approximately 90%. Fluorescence spectroscopy confirmed that the interaction between CA and Mb was driven by hydrogen bonding and van der Waals forces, resulting in a static quenching mechanism with a binding constant of 259.41 × 10^3^ L/mol at 293 K. Furthermore, UV–Vis and FT-IR spectroscopy indicated structural adjustments in Mb upon CA binding. Differential scanning calorimetry (DSC) showed an increased thermal denaturation temperature. Atomic Force Microscopy (AFM) showed CA-induced Mb aggregation. Consistently, molecular docking and MD simulations revealed that CA binds to the Mb molecule primarily through non-covalent interactions, such as hydrogen bonding. Collectively, these findings provide a theoretical basis for the application of CA as a natural color stabilizer in meat products.

## 1. Introduction

Meat color is a crucial aspect among the various qualities of meat, serving as a significant indicator for consumers when making purchasing decisions [1]. Consumers often associate meat color with its overall quality, such as microbial content or freshness, although it is not a highly accurate predictor of microbial levels per se [2]. A bright red color typically promotes sales, while color deterioration can lead to significant sales challenges [3]. It had been reported that the economic losses attributed to meat discoloration in the United States exceed $1 billion annually [1].

The color of meat from normally slaughtered livestock is primarily determined by the content and chemical state of myoglobin in the muscle. In post-slaughter meat, myoglobin typically exists in three forms: deoxymyoglobin, oxymyoglobin, and MetMb. Deoxymyoglobin, with iron in the ferrous state (Fe^2+^) and not bound to oxygen, gives meat a purplish-red color. Upon exposure to oxygen, it converts to oxymyoglobin (Fe^2+^-O_2_), producing a bright red color that consumers prefer. However, further oxidation converts the iron to the ferric state (Fe^3+^), forming MetMb, which gives meat a brown color that consumers often perceive as indicative of lower quality or spoilage [4,5]. To maintain the bright red color of fresh meat, meat scientists employ various strategies aimed at minimizing the relative proportion of MetMb formation in the meat [6].

A variety of meat color protection strategies have been extensively employed, which can be broadly categorized into physical and biochemical techniques. Among physical strategies, packaging technology—including vacuum packaging, modified atmosphere packaging (MAP), and active packaging—serves as a conventional approach for maintaining meat color. Vacuum packaging, by creating an oxygen-barrier environment, effectively inhibits the growth of aerobic microorganisms and extends shelf life [7]. However, the hypoxic conditions promote the accumulation of deoxymyoglobin, resulting in an unappealing purplish-red hue. In contrast, MAP with high oxygen levels (typically 70–80% O_2_) and controlled carbon dioxide facilitates the formation of oxymyoglobin, imparting a desirable bright red color that is attractive to consumers. Additionally, the incorporation of carbon monoxide in MAP can lead to the formation of carboxymyoglobin, which also exhibits a stable cherry-red color [8]. Advanced active packaging systems further enhance color stability by utilizing smart coatings or films that regulate gas exchange, provide antimicrobial effects, or even incorporate freshness indicators [9]. Several other physical processing methods also significantly influence meat color. For example, high-pressure processing may induce discoloration by promoting the formation of MetMb through protein denaturation or heme iron oxidation [10]. Illumination, particularly under specific wavelengths, can help delay MetMb formation and thus contribute to color stability [11]. On the other hand, cold atmospheric plasma treatment—which generates reactive oxygen and nitrogen species—may initially cause no immediate color change but can lead to gradual discoloration over time due to oxidative reactions [12].

Several small-molecular salt compounds are known to exert a color-protective effect in meat products, though their underlying mechanisms vary significantly. Sodium chloride stabilizes meat color predominantly by enhancing the structural stability of myoglobin [13]. In contrast, sodium nitrite coordinates with myoglobin to generate pink-colored nitrosylmyoglobin, thereby imparting color stability [14]. Meanwhile, intermediates of the tricarboxylic acid cycle—such as succinate, lactate, and pyruvate—contribute to color preservation primarily through enzymatic reactions that produce NADH [15,16]. This cofactor subsequently facilitates the reduction of MetMb to deoxymyoglobin, aiding in the maintenance of acceptable meat color [17,18]. In recent years, plant extracts have gained increasing attention as natural antioxidants for mitigating myoglobin oxidation induced by lipid and protein radicals. As the primary bioactive constituents of these extracts, polyphenols contribute to meat color preservation through multiple mechanisms, including free radical scavenging and transition metal chelation, which collectively suppress the oxidation of myoglobin and facilitate the reduction of MetMb to its functional ferrous form [5]. Several polyphenols—such as catechin, chlorogenic acid, gallic acid, ferulic acid, quercetin, and isoquercitrin—have been shown to enhance color stability by concurrently inhibiting lipid peroxidation and protein oxidation [3,19,20]. Upon binding to myoglobin, polyphenols may protect the heme pocket from oxidative attack by reducing solvent accessibility and restricting the conformational flexibility of the proximal/distal histidine residues, even under moderate conformational compaction. Conversely, extensive denaturation or non-specific aggregation would expose the heme iron, thereby accelerating oxidation. These activities help delay the formation of MetMb, thereby preserving the desirable red hue of meat products. Given their dual antioxidant and pro-reductive functions, plant-derived polyphenols represent a promising and sustainable strategy for maintaining meat color quality during processing and storage.

Caffeic acid, chemically known as 3,4-dihydroxycinnamic acid, is a natural phenolic compound belonging to the hydroxycinnamic acid family [21]. Its molecular core consists of three parts: a benzene ring, a vinyl group (forming an acrylic acid side chain), and a carboxyl group (as shown in Figure 1). The most prominent structural feature, which underlies its potent biological activities, is the presence of two adjacent hydroxyl groups (3, 4-dihydroxy substitution, constituting a catechol structure) on the benzene ring. This specific structure enables it to effectively scavenge free radicals and chelate metal ions, thereby inhibiting oxidation reactions [22,23].

Previous studies have indicated that CA can help stabilize meat color, primarily through mechanisms such as quenching free radicals, reducing MetMb, and inhibiting the activity of lipoxygenase (LOX) [5]. Research has confirmed that CA effectively scavenges H_2_O_2_, preventing it from generating highly reactive hydroxyl radicals via the Fenton reaction. Its ortho-dihydroxy structure gives it superior hydrogen peroxide-scavenging activity compared to other phenolic acids (e.g., p-coumaric acid). In meat models, CA can reduce lipid peroxidation indicators (such as TBARS values) and delay rancidity [5]. In the presence of reducing agents (e.g., cysteine), CA can directly reduce MetMb to oxymyoglobin (MbO_2_), thereby improving meat color stability. Experiments have shown that CA effectively maintains the red color (a* value) in meat emulsion systems [24,25]. Furthermore, CA inhibits LOX activity, possibly by chelating the iron ion in the enzyme or binding to its active site, which reduces the generation of volatile off-flavor compounds [26].

Although several studies have investigated the interactions between myoglobin and various polyphenols—including chlorogenic acid [27], quercetin [28] and (-)-epigallocatechin-3-gallate [3]—the specific non-covalent interaction mechanism between CA and myoglobin, and its potential implications for meat color stability, has not been systematically characterized. A more comprehensive literature review has been added to properly position this work within the existing body of knowledge. We hypothesized that CA interacts with myoglobin through non-covalent binding, inducing conformational changes that enhance the structural stability of the protein and thereby contribute to the preservation of meat color during refrigerated storage. Furthermore, we hypothesized that this interaction involves specific binding sites that can be characterized through integrated spectroscopic and computational approaches. The primary objective of this study is to explore the effects of different concentrations of CA on the conformation and microstructure of Mb. Furthermore, the binding process between myoglobin and CA will be examined, and their interaction forces and binding sites will be predicted using molecular simulation techniques.

## 2. Materials and Methods

### 2.1. Materials

The beef samples were obtained from the farmers’ market at Yantai University. The longissimus dorsi muscle was harvested from three Simmental crossbred steer carcasses at 3 days postmortem. The selected meat exhibited normal color, had pH values between 5.61 and 5.77, and showed no apparent quality defects.

The commercial CA (CAS No. 331-39-5, 98%) and Mb from equine skeletal muscle (CAS No. 100684-32-0, >95%) were purchased from Shanghai Yuanye Bio-Technology Co., Ltd. (Shanghai, China). The commercial bomophenol blue (BPB) were obtained from Shanghai Aladdin Biochemical Technology Co., Ltd. (Shanghai, China). All reagents employed in this study were of analytical grade, and ultrapure water was utilized for all experiments.

### 2.2. Methods

#### 2.2.1. Storage Experiment on the Effect of CA on the Color Stability of Beef

##### Sample Collection and Experimental Design

The experimental samples were produced by Yangxin Huasheng Halal Meat Co., Ltd. (Yangxin, Shandong, China). Three Simmental crossbred steer carcasses (24 months of age) were selected for this experiment. At 72 h post-mortem, the longissimus dorsi muscles were excised from the 12th to 13th rib section of each carcass. Visible surface fat and connective tissue were removed, and four steaks (5 cm × 5 cm × 2 cm) were prepared per carcass, yielding a total of 12 steaks. These steaks were randomly assigned to three treatment groups, with each carcass serving as an independent biological replicate (*n* = 3). Specifically, one steak per carcass was allocated to each of the following treatments: a control group, a 0.05% CA treatment, and a 0.1% CA treatment. The remaining steak from each carcass was used for initial baseline measurements.

##### Treatment Application and Storage Conditions

The CA treatments were applied via an immersion method. Steaks were immersed in their respective treatment solutions at a meat-to-solution ratio of 1: 5 (*w*/*v*) for 2 h. The treatment solutions consisted of deionized water (control), 0.05% CA, and 0.1% CA, with initial pH values of 7.0, 5.1, and 4.8, respectively. After immersion, the steaks were drained for 15 min. The final surface pH values of the steaks were 5.7 ± 0.1 (control), 5.6 ± 0.1 (0.05% CA), and 5.5 ± 0.1 (0.1% CA), with a weight change of less than 1.5%. The treated steaks were placed on sterile trays, overwrapped with polyvinyl chloride (PVC) film (oxygen transmission rate: 5000–8000 cm^3^/m^2^/24 h), and stored at 4 °C under 85–90% relative humidity and 1000 lux cool white fluorescent lighting on a 12-h light/12-h dark cycle. To ensure uniform light exposure, the positions of the trays were rotated daily throughout the 7-day storage period. Photographs of the meat pieces from all three groups were taken on storage days 0, 1, 3, 5, and 7. Additionally, the color parameters of the beef samples, including lightness (L*), redness (a*), and yellowness (b*), were measured using a CS-800 colorimeter obtained from Hangzhou Color Spectrum Technology Co., Ltd., (Hangzhou, China) under a D65 light source.

#### 2.2.2. CA-Mb Complex Solution

To form the Mb-CA complex for subsequent analysis, Mb was first dissolved in phosphate-buffered saline (PBS; 20 mM, pH 6.8) at a concentration of 1 mg/mL. This Mb solution was then incubated with varying final concentrations of CA (0, 3.125, 6.25, 12.5, 25, 50, and 100 μM). The final concentration of Mb in the reaction mixture was 0.5 mg/mL.

#### 2.2.3. Determination of the Relative Content of MetMb

Using a UV–vis spectrophotometer to measure the absorbance at 525, 545, 565, and 572 nm, the relative content of MetMb was calculated using the Krzywicki formula [27].

MetMb(%) = (−2.514R1 + 0.777R2 + 0.800R3 + 1.098) × 100,

where R1, R2, and R3 represented the absorbance ratios of A572/A525, A565/A525, and A545/A525, in that order.

#### 2.2.4. Determination of Solubility

The Mb solution (1 mg/mL) was incubated with varying concentrations of CA at 4 °C for 60 min, followed by centrifugation at 10,963× *g* for 15 min to separate the supernatant. The protein content in the resulting supernatant was then determined. The solubility (%) was calculated as the ratio of the protein concentration in the supernatant to the initial protein concentration. The solubility determination method has been fully described: protein concentration was quantified using the Bradford assay with bovine serum albumin (BSA) as the standard. The calibration curve was constructed over the range of 0.05–1.0 mg/mL (R^2^ = 0.998). The linear range was verified for each measurement batch. Potential interference from CA was assessed by measuring CA-only controls at each concentration; no significant interference was detected at the CA concentrations used in this study (absorbance at 595 nm < 0.02 for CA-only blanks). The corresponding blank (buffer without protein) was used for zero-point calibration. All measurements were performed in triplicate (n = 3 independent preparations).

Solubility% = (Mb content in supernatant/Total Mb content) × 100%

#### 2.2.5. Determination of Surface Hydrophobicity

To 1 mL of the Mb-CA complex solution (containing 0.5 mg/mL myoglobin), 200 μL of a 0.1 mg/mL bromophenol blue (BPB) solution was added. The mixture was vortexed thoroughly and then centrifuged twice (2000× *g*, 10 min). The absorbance of the resulting supernatant was measured at 595 nm. The amount of bound BPB, quantified based on the absorbance, was used as a hydrophobic index and calculated primarily according to the following formula.

BPB bound (μg) = 20 μg × (Acontorl − Asample)/Acontorl

#### 2.2.6. Differential Scanning Calorimetry to Assess Thermal Stability

The thermal stability of the Mb-CA complex was evaluated using a Q2000 Differential Scanning Calorimeter (TA Instruments, New Castle, DE, USA). An aliquot (2–6 mg) of the freeze-dried Mb-CA complex powder was accurately weighed and transferred into a sample pan, with an empty pan used as the reference. After ensuring proper data recording readiness, the sample was hermetically sealed. The heating rate was set at 5 °C/min, and DSC thermograms were acquired over a temperature range of 30–150 °C. Parameters including the onset temperature (T_0_), peak temperature (T_max_), and enthalpy of gelation (peak area; ΔH) for each sample were retrieved or calculated using the TA Universal Analysis software (Advantage v5.5.24).

#### 2.2.7. Fourier Transform Infrared Spectroscopy

Fourier transform infrared (FT-IR) spectroscopy was performed using an IRtrace-100 spectrometer (Shimadzu, Kyoto, Japan). The freeze-dried powder of the Mb-CA complex was mixed with potassium bromide (KBr) at a weight ratio of 1:100. Using KBr as a blank background, the mixture was thoroughly ground into a homogeneous powder using an agate mortar. Data were acquired in the absorption mode with 64 scans collected for the background spectrum. The resolution was set at 0.25 cm^−1^, and the spectral range was set at 400–4000 cm^−1^. After all spectra were collected, the data analysis and visualization were performed using OriginPro (Version 2025, OriginLab Corporation, Northampton, MA, USA).

#### 2.2.8. Ultraviolet–Visible Absorption Spectrometry

A UV-2550 UV–Vis spectrophotometer (Shimadzu, Kyoto, Japan) was used to scan the resulting mixtures from 200 to 750 nm after the myoglobin solution (1 mg/mL) was incubated with different concentrations of CA at 4 °C for 60 min.

#### 2.2.9. Synchronous Fluorescence Spectrum Detection

The synchronous fluorescence spectra of the Mb-CA complex were measured using a 0.5 mg/mL myoglobin solution at wavelength intervals (Δλ) of 15 and 60 nm, with spectral data recorded over the range of 240–350 nm.

#### 2.2.10. Endogenous Fluorescent Spectrum Detection

Fluorescence spectral analysis of the Mb-CA complex (using 0.5 mg/mL myoglobin solution) was performed on an F-7000 fluorescence spectrophotometer (Hitachi, Tokyo, Japan). Measurements were conducted in a 10 mm path length quartz cuvette under constant temperatures of 293 K, 299 K, and 305 K. The excitation wavelength was set at 283 nm, with both excitation and emission slit widths fixed at 5 nm. Data acquisition rate was 600 nm/min. Emission spectra were recorded in the 290–550 nm range for subsequent analysis. The inner filter effect on the fluorescence data was corrected using the equation described in the reference [29]. The fluorescence quenching mechanism of Mb by CA was determined using the Stern–Volmer equation (Equation (1)). The binding constant (Ka) and number of binding sites (n) were calculated via the double logarithmic equation (Equation (2)).

F_0_/F = 1 + Kqτ_0_[Q] = 1 + K_SV_[Q] (1)

log[(F_0_ − F)/F] = logKa + nlog[Q] (2)

where:

F_0_ and F = fluorescence intensities of myoglobin in the absence and presence of CA at a concentration of [Q], respectively, both values were corrected for the inner filter effect.
K_SV_ = Stern–Volmer dynamic quenching constant,
Kq = bimolecular quenching rate constant,
τ_0_ = the average fluorescence lifetime of the biomacromolecule in the absence of the quencher, with a value of 10^−8^ s.

Thermodynamic parameters—Gibbs free energy change (ΔG), enthalpy change (ΔH), and entropy change (ΔS)—were derived from the Van’t Hoff equation (Equation (3)) and the thermodynamic relationship (Equation (4)). The calculated parameters provide key insights into the dominant forces driving the myoglobin-CA interaction.

lnKa = −ΔH/RT + ΔS/R(3)

ΔG = ΔH − TΔS(4)

where:

Ka = binding constant at the given temperature,
T = experimental absolute temperature (K),
R = universal gas constant (approximately 8.314 J·mol^−1^·K^−1^).

#### 2.2.11. Atomic Force Microscope Detection

The solutions of Mb-CA complexes at varying concentrations were diluted 100-fold with phosphate-buffered saline (20 mmol/L, pH = 6.8), dropped onto mica substrates, and allowed to dry. Surface microstructures were scanned using an Atomic Force Microscope (MultiMode 8-HR, Bruker, MA, USA). The first scan covered an area of 10 μm × 10 μm, followed by a second scan over a 3 μm × 3 μm region. The experimental results were analyzed using Gwyddion 2.43 software.

#### 2.2.12. Molecular Docking and Molecular Dynamics Simulation

The SDF format of CA (CID: 689043) was downloaded from the PubChem database (https://pubchem.ncbi.nlm.nih.gov/). The three-dimensional structure of myoglobin (PDB ID: 1YMB) was obtained from the RCSB PDB database (www.rcsb.org). Automated blind docking of the ligand and receptor was performed using the online CB-Dock2 tool (http://cadd.labshare.cn/cb-dock2/php/index.php, accessed on 20 April 2023). The resulting docking poses were subsequently analyzed and visualized using PyMol 3.1.8 and LigPlot+ v2.2.

The PDB file obtained from the docking result of CA with Mb the Protein–Ligand complex was uploaded to the WeMol platform (https://wemol.wecomput.com) via GMX2023 calculation process for molecular dynamics (MD) simulation. A fully automated MD simulation with a duration of 100 nanoseconds was performed. The MD simulation procedure was as follows: the system was set up in a dodecahedron box using the Amber03 force field, with the environmental pH value set to 7. To neutralize the system charge, Na^+^ and Cl^−^ ions were added after solvating all components in the TIP3P water model. The energy minimization (EM) stage was carried out using the steepest descent integrator and concluded when the maximum force fell below 10.0 kJ/mol. For both the NVT (constant number of particles, volume, and temperature) and NPT (constant number of particles, pressure, and temperature) equilibration phases, the velocity Verlet algorithm was employed as the integrator, with temperature coupling achieved using the V-rescale method and pressure coupling using the C-rescale method. Each equilibration phase consisted of an initial simulation of 100 ps. Notably, to prevent potential system instability, the Protein_MOL and Water and ions index groups were fitted independently during temperature coupling. The production MD phase used a time step of 2 fs, with a total simulation time of 100 ns. By recording the trajectory every 10 ps, a dataset containing 10,000 frames was obtained. A constant temperature of 298 K was maintained throughout the simulation. Following the equilibration simulation, a trajectory file named “path.txt” was generated. Subsequently, structural analysis was performed using built-in commands from the GROMACS software suite (version 2023.4), including calculations of root-mean-square deviation (RMSD), root-mean-square fluctuation (RMSF), radius of gyration (Rg), and hydrogen bond analysis.

#### 2.2.13. Statistical Analysis

All experimental data were organized using Microsoft Excel, and are presented as the mean ± standard deviation (S.D.) (n = 3). Relevant graphs were plotted using Origin 2025 and GraphPad Prism 9 software. Statistical analysis was performed using IBM SPSS Statistics 26 software, employing one-way analysis of variance (ANOVA) followed by Tukey’s multiple comparison test to assess significant differences. Statistical significance was established at α = 0.05.

## 3. Result

### 3.1. Analysis of the Protective Effect of CA on Meat Color

Figure 2 depicts the color evolution of beef subjected to different treatments over a 7-day storage period. As shown, it demonstrated that all sample in three groups exhibited signs of color deterioration. However, the samples in control group underwent a significant color change, ultimately turning a purplish-brown. The samples in the groups treated with 0.05% and 0.1% CA solutions, localized browning occurred also, but the extent of discoloration was lighter compared to the control group.

The effects of different concentrations of CA on the color parameters (L*, a*, and b*) of beef steaks during 7 days of display are presented in Table 1.

Regarding lightness (L*), no significant differences (*p* > 0.05) were observed among the treatment groups throughout the storage period. The L* values remained relatively stable, ranging from approximately 42 to 45 across all samples, indicating that CA treatment did not significantly alter the surface brightness of the beef. In terms of redness (a*), which is a critical indicator of meat color stability, the CA-treated groups exhibited superior color retention compared to the control. On day 1, the a* values for the 0.05% and 0.1% CA groups (15.80 and 15.84, respectively) showed a slight increase compared to the control group (15.28), but these differences were not statistically significant (*p* > 0.05). This trend became more pronounced as display time increased. By day 5, the control group showed a marked decrease in redness (11.40), whereas the 0.05% and 0.1% CA treatments maintained significantly higher a* values (15.76 and 16.65, respectively). Although all groups experienced a decline in redness by day 7 due to oxidation, the CA-treated samples retained significantly higher redness (approx. 10.9) compared to the control (8.11). For yellowness (b*), the values generally decreased over time for all groups. However, on day 5 and day 7, the 0.1% CA treatment maintained significantly higher b* values compared to the control group.

Overall, the results suggest that CA treatment, particularly at 0.1%, effectively delays the discoloration of beef during retail display.

### 3.2. Analysis of MetMb%, Solubility Analysis, Surface Hydrophobicity Analysis, and DSC Analysis

As illustrated in Figure 3a., during the experimental process, compared with the samples in control group (without CA addition), the MetMb content of sample in treated groups exhibited an overall decreasing trend with increasing concentrations of CA (from 3.125 μM to 100 μM). Moreover, at CA concentrations ranging from 12.5 μM to 100 μM, the MetMb content was significantly lower than that of the control group (*p* < 0.05, 0.01, 0.001, 0.0001). These results indicate that the addition of CA at certain concentrations can inhibit effectively the accumulation of MetMb, which is consistent with the findings from the beef storage experiment.

The solubility of proteins is closely related to their functional properties. As shown in Figure 3b, the addition of CA resulted in a significant and dose-dependent inhibitory effect on the solubility of myoglobin.

In the control group without CA (0 μM CA), Mb maintained high solubility, approximately 90%. However, as the concentration of CA increased incrementally from 3.125 μM to 100 μM, the solubility of Mb exhibited a continuous declining trend. At the lowest tested concentration (3.125 μM), solubility began to decrease to approximately 85%. When the concentration reached 12.5 μM, solubility dropped to about 80%. Finally, at the highest tested concentration of 100 μM CA, the solubility of Mb was significantly reduced to approximately 60%. Furthermore, the differences in solubility of sample between all CA-treated groups and the control group (0 μM) were statistically extremely significant (*p* < 0.0001). These results suggest that CA molecules likely engage in specific interactions with Mb, altering the protein’s surface properties or spatial conformation.

Bromophenol blue is a classic hydrophobic fluorescent probe. Its binding amount with proteins can directly reflect the number and exposure degree of accessible hydrophobic regions or pockets on the protein surface. As shown in Figure 3c, the binding amount of BPB to myoglobin decreased significantly and monotonically in a dose-dependent manner with increasing concentrations of CA. In the control group (0 μM CA), the BPB binding amount reached its highest value, approximately 20 μg, representing the baseline exposure level of hydrophobic sites on the myoglobin surface in its natural state. Even with the addition of a low concentration of CA (3.125 μM), the BPB binding amount decreased significantly to about 15 μg. As the CA concentration increased multiplicatively, the binding amount continued to drop sharply: it decreased to approximately 10 μg at 6.25 μM, to about 8 μg at 12.5 μM, and further down to about 6 μg and 4 μg at 25 μM and 50 μM, respectively. Under the highest CA concentration of 100 μM, the BPB binding amount reached its lowest point, at merely about 2 μg, which represents a reduction of approximately 90% compared to the control group.

As shown in Figure 3d, the thermal denaturation curves of Mb and its mixtures with different concentrations of CA (3.125–100 μM) exhibit a prominent endothermic peak. With increasing CA concentration, this peak shows a systematic, dose-dependent shift towards higher temperatures. Specifically, the denaturation peak temperature (Tmax) of pure Mb is 82.37 °C, while upon addition of 100 μM CA, the Tmax increases significantly to 132.95 °C, representing a remarkable increase of 61.41%.

### 3.3. Fluorescence Spectroscopy Analysis

Figure 4a–c illustrates the fluorescence emission spectra of myoglobin (Mb) interacting with various concentrations of CA at different temperatures (293 K, 299 K, 305 K). The results show a characteristic fluorescence peak for Mb at approximately 340 nm. With the increase in CA concentration (from 0 to 100 µM), the fluorescence intensity of Mb decreases regularly (fluorescence quenching phenomenon) without significant peak shifting. This indicates that CA interacts with Mb, possibly forming a non-fluorescent complex or altering the conformation of Mb.

Figure 4d shows the Stern–Volmer plots, which exhibit good linearity. The bimolecular quenching rate constants (Kq) in Table 2 are on the order of 10^12^ L/(mol·s), which is much larger than the diffusion-controlled limit for biomacromolecules (2.0 × 10^10^ L/(mol⋅s)). This strongly proves that the fluorescence quenching of Mb by CA belongs to static quenching, meaning CA binds to Mb in the ground state to form a complex, rather than dynamic collisional quenching.

Table 2 shows that the binding constant (Ka) decreases significantly with increasing temperature (from 259.41 × 10^3^ at 293 K to 46.66 × 10^3^ at 305 K). This suggests that higher temperatures are unfavorable for complex formation, and the stability of the complex decreases as temperature rises. The number of binding sites (n) is close to 1, indicating that there is likely one primary binding site for CA on the Mb molecule.

Table 2 lists the thermodynamic parameters: Gibbs free energy change (ΔG) is negative, indicating that the binding process of CA to Mb is spontaneous. Both the enthalpy change (ΔH) and entropy change (ΔS) are negative. According to biochemical thermodynamic criteria, negative ΔH and ΔS typically indicate that hydrogen bonding and van der Waals forces are the main forces stabilizing the Mb-CA complex.

### 3.4. FTIR, UV–Vis Absorption Spectroscopy, and Synchronous Fluorescence Spectroscopy Analysis

Figure 5 illustrates the spectroscopic characteristics of the interaction between CA and myoglobin (Mb), specifically including Fourier Transform Infrared (FT-IR) spectroscopy, Ultraviolet–Visible (UV–Vis) absorption spectroscopy, and synchronous fluorescence spectroscopy.

Figure 5a presents the FT-IR spectra of Mb and Mb-CA complexes treated with varying concentrations of CA. The characteristic absorption peaks of Mb are primarily located in the Amide I band (approx. 1655 cm^−1^, corresponding to C=O stretching vibration) and the Amide II band (approx. 1547 cm^−1^, corresponding to C-N stretching and N-H bending vibrations). With the increasing concentration of CA (from 3.125 μM to 100 μM), slight red or blue shifts in peak positions and changes in peak intensities were observed in both the Amide I and Amide II bands. For instance, the Amide I band shifted gradually from 1655.97 cm^−1^ to approximately 1664.18 cm^−1^, with similar trends observed in the Amide II band. These variations indicate that the addition of CA altered the hydrogen bond network environment of the Mb secondary structure (primarily α-helices), suggesting that CA interacted with the carbonyl or amino groups on the Mb polypeptide backbone, leading to subtle conformational adjustments in the protein.

Figure 5b displays the UV–Vis absorption spectra of Mb complexed with different concentrations of CA. Mb exhibits a strong Soret band absorption peak at approximately 409 nm, which is characteristic of the heme prosthetic group, and a weaker absorption peak around 280 nm, primarily attributed to tyrosine (Tyr) and tryptophan (Trp) residues. As the concentration of CA increased, the absorbance of the Soret band at 409 nm decreased significantly (hypochromic effect), accompanied by a slight shift in peak position. This phenomenon suggests that CA may bind near the heme pocket of Mb or affect the polarity of the heme microenvironment through long-range interactions, thereby altering the electronic state of the heme iron and potentially influencing the oxygen-binding capacity or oxidative stability of Mb. Concurrently, the decrease in intensity at the 280 nm absorption peak further confirms the interaction between CA and the aromatic amino acid residues of Mb.

Figure 5c and d shows the synchronous fluorescence spectra at fixed wavelength intervals of Δλ = 15 nm and Δλ = 60 nm, respectively.

Δλ = 15 nm (Figure 5c): Under this condition, the synchronous fluorescence spectrum primarily reflects changes in the microenvironment of tyrosine (Tyr) residues. As shown, with increasing CA concentration, the fluorescence emission intensity of Tyr residues decreased significantly (fluorescence quenching), and the maximum emission wavelength underwent a slight blue shift (moving from approx. 307.1 nm towards shorter wavelengths). This blue shift indicates enhanced hydrophobicity and reduced polarity around the Tyr residues, implying that CA binding caused the Tyr residues to be more deeply buried within the hydrophobic cavities of the protein interior.

Δλ = 60 nm (Figure 5d): Under this condition, the spectrum mainly reflects changes in the microenvironment of tryptophan (Trp) residues. Similarly, a significant quenching of Trp residue fluorescence intensity was observed with increasing CA concentration, along with a blue shift in the maximum emission wavelength (shifting from approx. 338 nm to around 330 nm). This also indicates an increase in the hydrophobicity of the environment surrounding the Trp residues.

### 3.5. AFM Analysis

Figure 6 presents the two-dimensional (2D) and three-dimensional (3D) AFM topographical images of Myoglobin (Mb) and Mb-CA complexes adsorbed on mica surfaces under varying CA concentrations. These images aim to visually elucidate the impact of CA concentration on the aggregation state, surface roughness, and microstructural evolution of Mb molecules.

Figure 6a displays the AFM image of pure Mb samples in the absence of CA. The image reveals that Mb molecules are distributed relatively uniformly on the mica surface, appearing as dispersed globular or short rod-like particles with no obvious aggregates. The 3D image indicates a relatively smooth surface with a *Z*-axis height range of only 0–3.3 nm, suggesting that Mb exists primarily as monomers or very small oligomers with low surface roughness under these conditions.

Initial aggregation (Figure 6b–d) is induced by Low Concentrations of CA (3.125–12.5 μM CA); with the introduction and increase in CA concentration (Figure 6b–d), the micro-morphology of Mb undergoes significant changes. At 3.125 μM CA (Figure 6b), discrete granular protrusions appear on the surface, with the height increasing to 7.5 nm, indicating that CA initiates preliminary interactions or conformational changes in Mb molecules. As the CA concentration rises to 6.25 μM (Figure 6c) and 12.5 μM (Figure 6d), the density of surface particles increases markedly, and small-scale cluster aggregates begin to emerge. The 3D images show a sharp rise in surface roughness, with *Z*-axis heights reaching 12 nm and 23 nm, respectively. This suggests that CA molecules, acting as cross-linkers or mediators of hydrophobic interactions, promote intermolecular binding of Mb, leading to the formation of oligomers.

Large-scale aggregation and fibrillation (Figure 6e–g) induced by high concentrations of CA (25–100 μM), under high CA concentrations, the aggregation behavior of Mb intensifies further, resulting in the formation of more complex supramolecular structures.

At 25 μM CA (Figure 6e) and 50 μM CA (Figure 6f), a large number of larger spherical or irregular block-like aggregates appear in the field of view. In some regions, chain-like structures resembling protofibrils connecting the aggregates are even observed. The *Z*-axis heights increase to 28 nm and 34 nm, respectively. When the CA concentration reaches 100 μM (Figure 6g), the size of the aggregates increases further, forming dense stacks of large particles with a maximum height of 39 nm. This significant morphological change indicates that high concentrations of CA greatly enhance hydrophobic interactions or hydrogen bond network reorganization among Mb molecules, leading to severe protein aggregation or even precipitation, forming micro-aggregates visible on a macroscopic scale.

### 3.6. Molecular Docking and Molecular Dynamics Simulation Analysis

Molecular docking results indicate that CA embeds within the hydrophobic cavity of myoglobin (Mb) (Figure 7). The 2D interaction plot reveals that the binding is primarily stabilized by hydrogen bonds and hydrophobic interactions. Specifically, the hydroxyl groups of CA form stable hydrogen bonds with residues Lys87 (2.37 Å), Glu148 (2.86 Å), and Asn145 (3.00 Å). Furthermore, significant hydrophobic contacts and pi-alkyl interactions are observed between the benzene ring of CA and residues Ala81, His82, and Asp141, suggesting that hydrophobic effects are a major driving force for ligand entry into the binding pocket.

Trajectory analysis from 100 ns molecular dynamics (MD) simulations further validates the dynamic stability of the complex (Figure 8).

Structural Stability and Flexibility: The Root Mean Square Deviation (RMSD) curves show that the Mb-CA complex reaches equilibrium rapidly after an initial adjustment. The RMSD values of the complex are slightly lower than or comparable to those of pure Mb, indicating enhanced backbone stability upon CA binding (Figure 8a). Root Mean Square Fluctuation (RMSF) analysis demonstrates that, except for specific flexible loops, the fluctuation amplitude of residues in the complex is low, confirming that CA binding does not induce drastic conformational disruption (Figure 8b).

Interaction Persistence: The plot of hydrogen bond number versus time shows that a consistent number of hydrogen bonds are maintained throughout the simulation (Figure 8c), corroborating the stability of the hydrogen bond network identified in docking.

Compactness and Solvent Accessibility: The Radius of Gyration (Rg) curves indicate that the Rg of the complex remains constant and is slightly smaller than that of pure Mb (Figure 8d), suggesting that CA binding induces a more compact protein structure. Solvent Accessible Surface Area (SASA) analysis (Figure 8e) reveals that the total, hydrophobic, and hydrophilic surface areas remain stable, indicating good conformational stability in an aqueous environment.

Free Energy Landscape: The Free Energy Landscape (FEL) (Figure 8f) displays the potential energy surface distribution. The Mb-CA complex exhibits a deep and narrow global energy minimum, indicating that this binding conformation represents the most thermodynamically stable state.

## 4. Discussion

### 4.1. Mechanisms of CA in Inhibiting MetMb Formation and Maintaining Meat Color Stability

Results from both in situ and in vitro model experiments (Figure 2 and Figure 3a, and Table 1) consistently demonstrate that CA effectively mitigates MetMb accumulation and maintains meat color stability. These findings indicate that the addition of CA at specific concentrations can significantly inhibit MetMb accumulation, which is in good agreement with the results obtained from the beef storage assay. Furthermore, this observation aligns with previous studies [20,28,29], which attribute CA’s protective effects to its unique molecular structure. This structure endows CA with remarkable antioxidant activity, thereby preventing myoglobin oxidation and contributing to the maintenance of meat color stability.

It has been previously reported that the color-stabilizing effects of polyphenols, such as CA, are achieved primarily through three pathways:

Direct Scavenging of Free Radicals and Interruption of Oxidative Chain Reactions

The catechol moiety within the molecular structure of CA serves as the primary source of its potent antioxidant activity [20]. This functional group enables CA to efficiently scavenge reactive oxygen species (ROS), such as superoxide anions and hydroxyl radicals, generated within the meat matrix. By doing so, it directly interrupts the chain reactions of lipid peroxidation and myoglobin oxidation, thereby protecting the red oxymyoglobin from being oxidized into the brown MetMb.

Direct Interaction with Myoglobin and Structural Stabilization

Previous studies have demonstrated that CA can specifically bind to myoglobin [24]. This interaction confers two primary protective effects: Inhibition of MetMb Formation: CA can donate electrons to oxymyoglobin, thereby retarding its autoxidation process.

Stabilization of Myoglobin Conformation: By binding to specific amino acid residues, CA helps maintain the native three-dimensional conformation of myoglobin, preventing pigment degradation induced by oxidative stress or thermal denaturation.

Synergistic Reductive Effects

Under specific conditions (e.g., in the presence of reducing agents such as cysteine), CA exhibits enhanced reducing capacity. This allows it to directly reduce the already formed MetMb back to oxymyoglobin [30]. This dynamic equilibrium between discoloration and color restoration ultimately translates to superior macroscopic color stability.

### 4.2. Solubility, Surface Hydrophobicity, and DSC Discussion

The possible mechanisms for the decreased solubility of MB-CA include: the polyphenolic structure of CA binding to specific amino acid residues (such as lysine, arginine, or histidine) on the Mb surface, or engaging in hydrophobic interactions, which may neutralize partial charges on the protein surface or induce limited aggregation of protein molecules. Consequently, these interactions could reduce the hydrophilicity of Mb, ultimately leading to a decrease in its solubility in solution. Consistent findings have been reported in previous research regarding the interaction between chlorogenic acid and myofibrillar protein [31].

A balanced discussion addresses whether the observed reduction in Mb solubility represents a beneficial or undesirable effect: reduced solubility may indicate protein aggregation or precipitation, which could negatively affect meat texture and water-holding capacity; alternatively, reduced solubility driven by CA-induced conformational tightening may reflect enhanced structural stability that protects the heme environment. We discuss both perspectives and acknowledge that the net effect on product quality requires further investigation in actual meat products.

This result of surface hydrophobicity confirms a relatively strong interaction between CA and Mb. It demonstrates that CA can specifically bind to the hydrophobic regions of Mb, potentially through pathways such as competitive binding or induction of protein conformational changes, thereby significantly altering the surface hydrophobic properties of myoglobin. The observed changes in both hydrophobic interaction and solubility confirm the interaction between CA and Mb, which consequently modifies the protein’s solubility and hydrophobic behavior.

We provide a biological justification for the decreased BPB binding, considering multiple contributing factors: (1) conformational tightening that buries hydrophobic patches, reducing available BPB binding sites; (2) direct competitive binding between CA and BPB at shared hydrophobic sites on the Mb surface; (3) CA-induced aggregation that reduces the effective surface area available for BPB interaction. We acknowledge that the current experimental design cannot definitively distinguish among these mechanisms, and orthogonal approaches (e.g., isothermal titration calorimetry, competitive binding assays) would be needed.

The endothermic peak in the DSC curve corresponds to the cooperative unfolding (denaturation) process of the protein structure. The increase in Tmaxdirectly indicates enhanced thermal stability of the protein [32]. In the present research, the continuous and significant rise in Tmaxwith increasing CA concentration provides strong evidence that CA interacts with Mb and stabilizes its native three-dimensional structure in a dose-dependent manner, requiring a higher input of thermal energy to induce denaturation.

This enhancement in thermal stability typically originates from the binding of CA molecules to specific sites on the protein. Potential mechanisms include the following: (1) Non-covalent interactions: CA may bind to Mb via hydrogen bonding, hydrophobic interactions, or electrostatic forces, which partially ‘locks’ the protein conformation, increases its structural rigidity, and consequently raises the energy barrier (manifested as a higher temperature) for the unfolding process. (2) Complex formation: The formation of the CA-Mb complex may alter the protein’s solvation environment or internal packing, thereby enhancing its overall conformational stability.

The DSC data clearly demonstrate that CA significantly improves the thermal stability of Mb, and this effect is concentration-dependent. This provides key thermodynamic evidence for a direct physicochemical interaction between CA and Mb, indicating that CA binding effectively stabilizes the native conformation of Mb.

Correlation with Solubility Experiment Results: The solubility assay showed that CA causes a decrease in Mb solubility. Combined with the strong stabilizing effect revealed by DSC, it can be inferred that the CA-induced decrease in solubility is not primarily due to protein denaturation and aggregation. Instead, it is more likely a result of altered protein surface charge, polarity, or hydration layer following CA binding, which reduces its hydrophilicity. Importantly, the protein maintains a highly stable, folded state (or an even more stable one) in solution, but the nature of its interaction with the solvent has changed.

Correlation with surface hydrophobicity Results: The BPB assay indicated that CA binds to the hydrophobic regions of Mb and makes them ‘hidden’ (less accessible). The DSC results further confirm that this binding is not merely a superficial coverage but profoundly alters the overall energy landscape of the protein. By stabilizing the core folded structures where these hydrophobic regions reside, the binding substantially enhances the global stability of the protein.

### 4.3. Fluorescence Spectroscopy Discussion

Fluorescence spectroscopy serves as a powerful analytical tool for elucidating interaction mechanisms between functional small molecules and proteins, enabling the characterization of key parameters including quenching mechanisms (static and dynamic processes), Stern–Volmer quenching constant (K_SV_), quenching rate constant (Kq), affinity determinations (Ka), quantification of binding sites (n), and classification of interaction types [32,33,34]. This technique thereby provides insights into molecular recognition processes and binding dynamics at the biophysical level.

Tyrosine (Tyr), tryptophan (Trp), and phenylalanine (Phe) represent the primary sources of intrinsic fluorescence in proteins [35]. Mb, consisting of approximately 153 amino acid residues, forming a polypeptide chain, contains two Trp residues, two Tyr residues, and seven Phe residues. Therefore, the intrinsic fluorescence emitted by these aromatic amino acid residues can be utilized to investigate the interaction between CA and Mb through fluorescence quenching analysis. As illustrated in Figure 4a–c, the fluorescence emission profiles of Mb were systematically evaluated under three temperature gradients (293 K, 299 K, and 305 K) in the presence of escalating concentrations of CA (0–100 μM). A concentration-dependent reduction in Mb’s intrinsic fluorescence was observed, with the characteristic emission peak at approximately 329 nm exhibiting progressive attenuation as CA levels increased. The Stern–Volmer plots (Figure 4d; Table 2) revealed strong linear correlations across all temperature conditions, with Stern–Volmer quenching constants (K_SV_) of 8.33 × 10^4^ L/mol (R^2^ = 0.989), 6.04 × 10^4^ L/mol (R^2^ = 0.997), and 4.87 × 10^4^ L/mol (R^2^ = 0.996) determined at 293 K, 299 K, and 305 K, respectively. The observed decline in K_SV_ with rising temperature further supports the dominance of static quenching as the primary mechanism, indicating the formation of a stable Mb-CA complex in the ground state. For macromolecular systems, the theoretical upper limit for dynamic quenching constants via diffusive collisions typically does not exceed 2 × 10^10^ L/(mol·s). However, the calculated quenching rate constant (Kq) in this study significantly surpasses this threshold, reinforcing the conclusion that CA interacts with Mb through static complexation rather than transient collisional events [36]. These findings collectively demonstrate that CA binds to Mb with high affinity, inducing structural perturbations that modulate its fluorescence properties.

The binding affinity and stoichiometry of the Mb-CA interaction were determined through the double-logarithm method (Figure 4e, Table 2). Analysis of the Stern–Volmer data revealed strong linear fitting for the static quenching model at three temperatures, yielding association constants (K_a_) of 259.41 L/mol (R^2^ = 0.989), 24.13 L/mol (R^2^ = 0.979), and 6.66 L/mol (R^2^ = 0.987) at 293 K, 299 K, and 305 K, respectively. These K_a_ values exhibit a negative correlation with temperature, indicating a thermodynamic destabilization of the Mb-CA complex at elevated temperatures. This trend suggests that the binding process is enthalpically driven, with higher thermal energy disrupting the non-covalent interactions stabilizing the complex. The calculated binding site numbers (n) across all temperature conditions were approximately 1.0 (Table 2), confirming a 1: 1 stoichiometric ratio between Mb and CA. This implies the presence of a single, specific binding site on Mb for CA, consistent with a site-selective interaction mechanism. The temperature-dependent reduction in K_a_ further supports the hypothesis that hydrophobic forces or hydrogen bonding—commonly sensitive to thermal perturbations-predominate in Mb-CA complexation [27,36].

To elucidate the dominant forces governing Mb-CA complexation, thermodynamic parameters were derived from binding data (Table 2). Non-covalent interactions—including hydrogen bonding, hydrophobic forces, van der Waals attractions, and electrostatic forces—typically induce characteristic changes in enthalpy Gibbs free energy (ΔG), (ΔH), and entropy (ΔS) [37]. The negative ΔG value validates spontaneous complexation, aligning with prior studies on bovine lactoferrin–ginsenoside Rg3 binding [38]. Notably, the magnitude of ΔG suggests high binding affinity and thermodynamic stability, as smaller absolute ΔG values correlate with enhanced spontaneity and complex stability [39]. The calculated thermodynamic profile revealed ΔH = −227.14 kJ/mol (exothermic), ΔS = −0.672 J·mol^−1^·K^−1^ (entropy-decreasing), respectively. Thus, the ΔH and ΔS values were all < 0 which suggested that the main forces acting between CA and Mb were van der Waals and hydrogen bonds. This result is consistent with other studies that have reported that the driving forces for the binding of phenolic compounds (ferulic acid, chlorogenic acid, and morin) to globular proteins (β-lactoglobulin, tyrosinase) were mainly van der Waals forces and hydrogen bonds [40,41].

### 4.4. FTIR, UV–Vis Absorption Spectroscopy, and Synchronous Fluorescence Spectroscopy Discussion

#### FTIR Discussion

Fourier-transform infrared spectroscopy (FT-IR) is widely employed to characterize polymer composition and molecular interactions, including protein-based systems [42]. Figure 5a presents the key characteristic peaks of the myoglobin spectrum, with the amide A band (3300–3450 cm^−1^) exhibiting a peak at 3332.84 cm^−1^, the amide B band at 2978.52 cm^−1^, the amide I band (1600–1700 cm^−1^) at 1655.97 cm^−1^, and the amide II band (1530–1550 cm^−1^) at 1547.01 cm^−1^. The amide A, amide B, and amide I bands are primarily associated with C=O stretching vibrations, while the amide II band corresponds to C-N stretching and N-H bending vibrations [42,43,44]. The FT-IR spectrum of myoglobin treated with CA was generally consistent with that of the control group, with no significant alterations observed in the overall peak profile. However, CA treatment distinctly influenced both the peak positions and peak intensities of myoglobin. Specifically, the characteristic peaks of CA-treated myoglobin exhibited shifts toward higher wavenumbers (blue shift), and their intensities varied in a concentration-dependent manner with increasing CA levels. These findings are generally consistent with the study by Sun et al. [28].

Specifically, at CA concentrations of 3.125, 6.25, 12.5, 25, 50 and 100 μM, the amide A band exhibited shifts of 23.14, 39.56, 26.12, 36.57, 20.90, and 14.93 cm^−1^, respectively; the amide B band shifted by 14.47, 23.14, 2.43, 24.59, 7.23, and 7.72 cm^−1^; the amide I band shifted by 5.22, 5.22, 8.21, 10.45, 2.99, and 8.21 cm^−1^; and the amide II band shifts were equal to or greater than those observed for the amide I band, with measured values of approximately 5.22, 11.22, 5.22, 2.98, and 2.98 cm^−1^, respectively. These results indicate that CA induced conformational changes in the secondary structure of Mb and formed non-covalent interactions with the protein, such as hydrogen bonding [42]. These findings are consistent with the obtained thermodynamic parameters.

UV–vis absorption spectroscopy discussion

Changes in the intensity and location of peaks in ultraviolet–visible (UV–Vis) absorption spectra provide critical insights into protein–ligand interactions and the associated conformational changes in proteins. These spectral alterations reflect modifications in the microenvironment surrounding chromophores, such as aromatic amino acid residues and the heme group [45]. In the absorption spectrum of myoglobin (Mb), two prominent peaks are typically observed: the peak around 280 nm, corresponding to π → π* electronic transitions of aromatic amino acids like tyrosine (Tyr) and tryptophan (Trp), and the Soret band around 409 nm, which arises from the heme–globin interaction [46,47].

As shown in the UV–Vis absorption spectra of Mb treated with different CA concentrations (Figure 4b), the addition of CA resulted in a* significant decrease in the absorbance of the Soret band at 409 nm (hypochromic effect), accompanied by a slight shift in peak position. This hypochromicity suggests that CA interacts with Mb in the vicinity of the heme pocket or influences the polarity of the heme microenvironment through long-range interactions. Such interactions alter the electronic state of the heme iron, which may have implications for the oxygen-binding capacity or oxidative stability of Mb.

Furthermore, the absorption peak around 280 nm exhibited a decrease in intensity with increasing CA concentration. This reduction indicates that CA interacts with the aromatic amino acid residues of Mb, leading to a change in their microenvironment. Combined with the blue shift observed in synchronous fluorescence spectra, this decrease in absorbance suggests that the binding of CA induces conformational changes that bury the aromatic residues in a more hydrophobic environment (lower polarity) or involves the formation of hydrogen bonds that perturb the electronic transitions of these residues [30].

Collectively, the variations in the UV–Vis spectra—specifically the hypochromic effect at 409 nm and the intensity reduction at 280 nm—provide strong evidence for the formation of a ground-state complex between CA and Mb. This observation supports the conclusion that the fluorescence quenching mechanism is static quenching rather than dynamic quenching [37], as dynamic quenching typically affects the excited state without altering the absorption spectrum of the ground state.

Synchronous fluorescence spectroscopy discussion

The spectral features of chromophores with different properties are reflected in the difference between excitation and emission wavelengths, Δλ (Δλ = λem − λex). Protein fluorescence is primarily attributed to the tyrosine (Tyr) residue when the Δλ value is 15 nm, whereas its association with Trp residues is suggested when the Δλ value is 60 nm [48]. The maximum absorption wavelength associated with Trp and Tyr residues showed a slight redshift following CA treatment (Figure 5c,d). This implies that Tyr and Trp residues are exposed to a hydrophobic aqueous solution and that polarity has increased [49]. In the meantime, the fluorescence intensity of Trp residues was greater than that of Tyr residues, suggesting that Trp residues had a better quenching effect on the endogenous fluorescence of myoglobin. The fluorescence intensity of both Tyr and Trp residues clearly decreased as the CA concentration rose. This demonstrated that CA successfully quenched the fluorescence of myoglobin and created a stable complex with it [50].

The aforementioned spectroscopic results consistently demonstrate that CA binds to Mb through non-covalent interactions (such as hydrogen bonding and hydrophobic interactions). This binding not only reduces the polarity (increases hydrophobicity) of the microenvironment of surface aromatic amino acids (Tyr and Trp), leading to a more compact protein conformation or altered local folding, but also affects the electronic environment of the internal heme prosthetic group. These molecular-level interactions likely constitute the physicochemical basis for CA’s ability to stabilize the Mb structure and inhibit its oxidative discoloration.

### 4.5. AFM Discussion

The AFM is a type of scanning probe microscope that was created using the same principles as a scanning tunneling microscope. It accurately determines the shape and geometry of proteins and allows atomic-scale surface measurements without altering their original structure [51,52]. The AFM images of myoglobin, encompassing both two-dimensional and three-dimensional representations, are presented in Figure 6. The AFM images of Mb in the control group was smaller and spherical in shape. But when CA was added, Mb showed a remarkable crosslinking effect that clearly caused aggregation. As the CA concentration rose, Mb aggregation became more noticeable, and the aggregates’ surfaces became noticeably rough. Furthermore, the molecular height tended to increase while the myoglobin molecules showed increased swelling and size. According to the findings, CA causes myoglobin crosslinking, which modifies its structure and causes it to aggregate. This may be due to the various molecular forces that are produced by their interactions [53,54,55]. Consequently, the addition of CA to proteins may change how protein chains interact and promote the formation of protein–CA complexes.

### 4.6. Molecular Docking and Molecular Dynamics Simulation Discussion

The overall conformational stability of the Mb-CA complex is enhanced compared to that of free Mb, as revealed by RMSD and FES analysis. RMSD is a key metric used to evaluate the average deviation of the entire protein backbone relative to its initial structure [56,57]. As shown in Figure 8a, the RMSD value of the Mb-CA complex (blue line) remains stable at approximately 0.1 nm throughout the simulation period, whereas the free Mb (red line) exhibits greater fluctuations, ranging from 0.15 to 0.25 nm. This indicates that CA binding effectively restricts the overall conformational drift of the protein and significantly enhances its global rigidity.

As depicted in Figure 8f, the three-dimensional FEL, plotted using RMSD and Rg as reaction coordinates, visually represents the relative free energy of the system across distinct conformational states. The FEL reveals that the Mb-CA complex exhibits a deep and localized global free energy minimum (purple basin) with a depth of approximately −40 kJ/mol, contrasting sharply with the shallow and broadly distributed energy wells observed for free Mb.

Thermodynamically, this signifies the presence of a dominant, highly stable low-energy conformational state in the Mb-CA complex, where the system is strongly stabilized [58,59]. This finding aligns with the reduced RMSD fluctuations detected in the trajectory analysis, further confirming the enhanced structural rigidity induced by CA binding.

Structural Compaction and Local Flexibility Modulation Analysis (Based on Rg and RMSF). The Rg values were displayed in Figure 8d. This parameter reflects the mass distribution of the protein, indicating its overall compactness [60]. The Rg values of the Mb-CA complex are consistently lower than those of free Mb, suggesting that CA binding induces a global structural contraction, rendering the protein more compact. This could arise from ligand-induced conformational adjustments or direct cavity-filling effects within the protein interior. Figure 8b showed the RMSF values. This metric reflects the local flexibility of individual amino acid residues [61]. Although the overall RMSF profiles for both the complex and free protein are similar, indicating maintained stability in the core secondary structure regions, closer inspection reveals differences in fluctuation amplitudes for specific residue ranges (e.g., certain loop regions) in Mb-CA compared to free Mb. This suggests that CA binding may specifically influence the local dynamics of key residues near the binding pocket or within potential allosteric pathways. Such fine-tuning is likely integral to achieving stable binding and may relate to functional modulation.

Remodeling of the Intra-Protein Interaction Network and Surface Properties of Mb-CA Analysis (Based on Hydrogen Bond and SASA). According to Figure 8c, during the 100-ns simulation, the number of hydrogen bonds between the Mb-CA complex fluctuates between 0 and 6. This range is common and reasonable for protein-ligand complexes, indicating that CA formed a moderate number of dynamically fluctuating hydrogen bonds with Mb. The bond count is neither too low (indicating weak binding) nor excessively high (which might lead to overly rigid binding modes), thereby providing a foundation for stable and specific interactions [62]. This time-dependent hydrogen bond count plot clearly reveals the binding dynamics of the Mb-CA complex: it transitions rapidly from an initial unstable and rapid exploration phase to a dynamically balanced hydrogen bond interaction network. The sustained presence of several stable hydrogen bonds in the later stages serves as a critical microscopic basis for the structural stability of the complex. This observation corroborates the macroscopic conclusion derived from RMSD and FES analyses—that CA binding enhances the overall stability of Mb. The SASA results are presented in Figure 8e, The SASA analysis reveals profound effects of CA binding on the surface properties of the protein [60]. The total SASA remains relatively stable, indicating no significant global size changes. This aligns with the slight structural compaction observed in the radius of gyration (Rg) analysis. The hydrophobic SASA of the Mb-CA complex is significantly lower than that of free Mb. This serves as one of the most direct pieces of evidence for CA binding, demonstrating that the CA molecule occupies and shields hydrophobic patches or pockets on the Mb surface, rendering them less accessible to the solvent. The hydrophilic SASA of Mb-CA is slightly higher compared to free Mb. This may be attributed to a conformational compensation effect driven by structural compaction and the burial of hydrophobic regions, which enhances surface hydrophilicity to maintain solubility [56].

Molecular dynamics simulations systematically reveal that CA, as a ligand, binds to myoglobin Mb through a complex, multi-faceted process: Thermodynamic driving force: The binding establishes a stable complex conformation with a substantially lower free energy, as demonstrated by the free energy surface (FES) analysis. Structural effects: This conformation exhibits reduced global flexibility (lower RMSD) and increased compactness (reduced Rg). Interaction mechanism: CA primarily binds by occupying and shielding hydrophobic surface regions of Mb (decreased hydrophobic SASA), accompanied by a reorganization of the protein’s intramolecular hydrogen bond network (reduced intramolecular hydrogen bonds). This binding mode stabilizes the protein structure while altering its surface properties (elevated hydrophilic SASA). Functional implications: Such stabilization and surface modification may modulate Mb’s oxygen-binding capacity, antioxidant potential, or interactions with other molecules. These findings provide molecular-level insights into how small ligands (e.g., polyphenolic compounds) regulate the structure and function of globular proteins. In summary, the simulation data collectively and consistently support the core conclusion that CA induces Mb to adopt a more stable and compact conformational state through specific binding interactions.

However, we note potential discrepancies: the van’t Hoff-derived ΔH value may reflect contributions from conformational changes and coupled protonation events that are not captured by the MD simulation. The free-energy landscape projected from RMSD and Rg provides relative conformational populations but should not be interpreted as the absolute binding free energy. Rigorous binding free-energy calculations (e.g., MM/PBSA or MM/GBSA) would be required for quantitative comparison, and this is noted as a priority for future work.

Molecular docking and molecular dynamics simulations generate mechanistic hypotheses based on computational models and force-field approximations. These approaches do not experimentally prove the binding mechanism or binding site. The predicted binding pose and key interaction residues should be validated through site-directed mutagenesis, NMR titration, or X-ray crystallography of the Mb–CA complex. Furthermore, the MD simulation was limited to a single 100-ns trajectory per system, which may not fully capture the conformational ensemble. Independent replicate simulations would be required to establish robust statistical differences in dynamic behavior.

## 5. Conclusions

This study comprehensively demonstrates that CA binds to Mb through non-covalent interactions, including hydrogen bonds, hydrophobic forces, and electrostatic interactions, leading to structural and functional modulation of Mb. Key findings include:

In meat storage experiments, 0.1% CA effectively preserved the fresh red color (a* value) of meat and delayed browning. The underlying mechanism likely involves scavenging free radicals and reducing lipid peroxidation. This dual antioxidant and reducing activity correlates with CA-induced structural modifications of myoglobin (Mb), specifically the stabilization of its ferrous state. CA protected hydrophobic regions (90% reduction BPB binding) while enhancing hydrophilicity, thereby altering Mb’s solvation properties. This surface structural reorganization is associated with CA’s ability to inhibit MetMb formation (up to a 6% reduction), thereby maintaining the vibrant red color of meat during storage. Fluorescence spectroscopy and molecular docking analyses identified specific binding residues (e.g., Glu148, Asn145, and His82) and confirmed an approximate 1: 1 molar stoichiometry, with van der Waals forces and hydrogen bonds as the dominant driving forces. This binding alters local flexibility in loop regions, potentially modulating allosteric pathways critical to Mb function. Furthermore, CA enhanced Mb’s overall rigidity, as evidenced by reduced RMSD: 0.1 nm and improved thermal stability (Tmax = 132.95 °C at 100 μM CA). Molecular dynamics simulations revealed that CA restricts conformational drift, stabilizing a compact structure with lower free energy (−40 kJ/mol), consistent with experimental thermodynamic data (ΔG < 0).

The study establishes a molecular framework for polyphenol-based strategies in meat color preservation, highlighting CA’s potential to replace synthetic additives through natural, multifunctional mechanisms. By bridging structural biology and food science, these findings advance understanding of how small molecules regulate globular protein functionality, offering a blueprint for developing sustainable meat color preservation technologies. Therefore, the present study highlights the significance of protein-polyphenol interactions in regulating meat color and provides practical recommendations for the potential application of plant-derived antioxidants in meat systems.

While the present study provides preliminary evidence for the potential of CA as a natural color preservative for meat, several critical experiments are required before practical application can be recommended: (1) sensory evaluation to assess the impact of CA treatment on meat flavor, odor, and consumer acceptability; (2) toxicological safety assessment at the effective concentrations; (3) evaluation under realistic processing conditions, including cooking, freezing–thawing, and modified atmosphere packaging; (4) direct measurement of MetMb content, lipid oxidation (TBARS), and microbial status in CA-treated beef to establish the causal link between the proposed molecular mechanisms and observed color preservation; (5) dose–response studies bridging the concentration gap between the molecular experiments (μM) and the meat application (mM); and (6) investigation of CA in combination with other natural preservatives to optimize formulation.

## Figures and Tables

**Figure 1:**
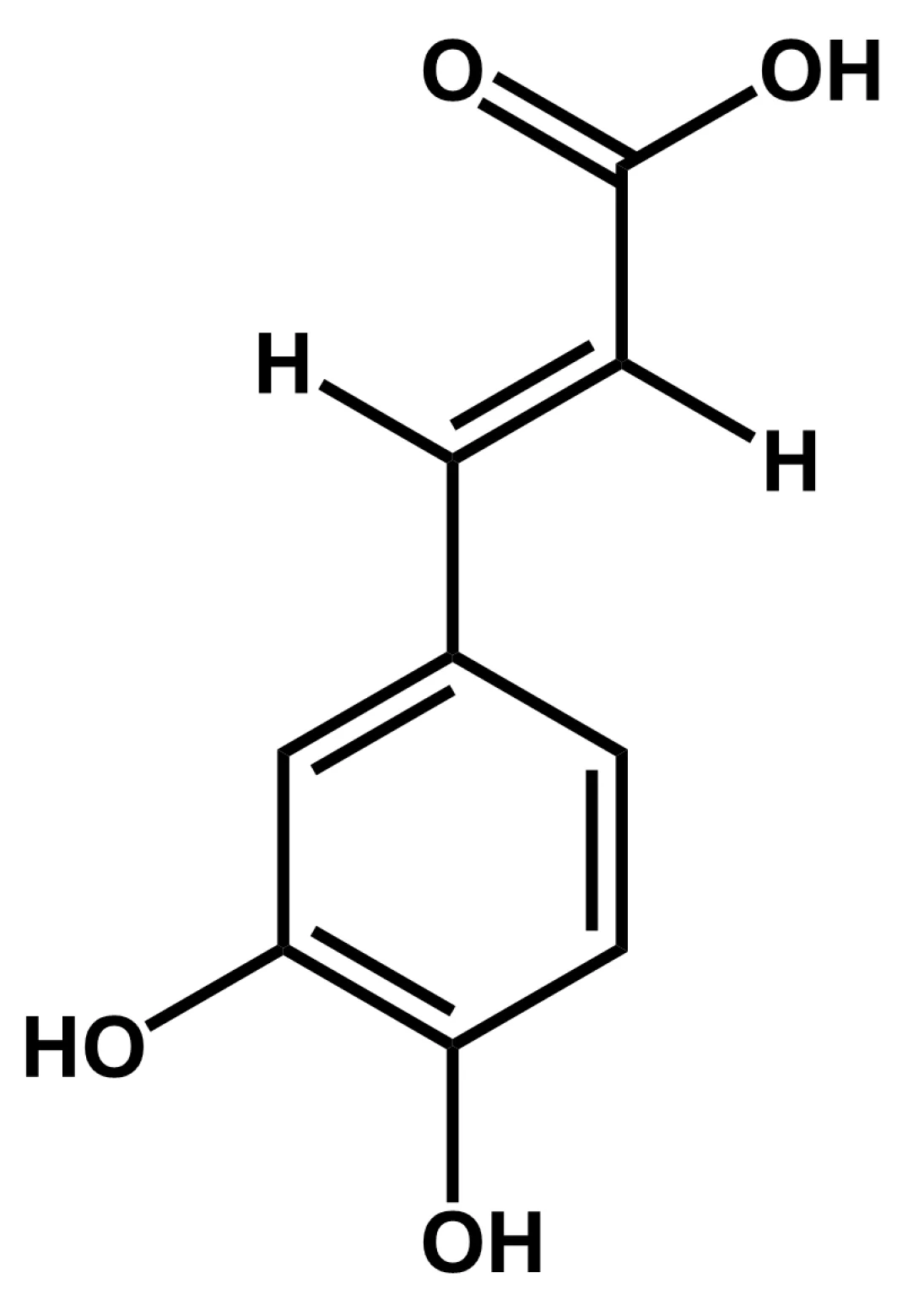
Chemical structure of CA.

**Figure 2:**
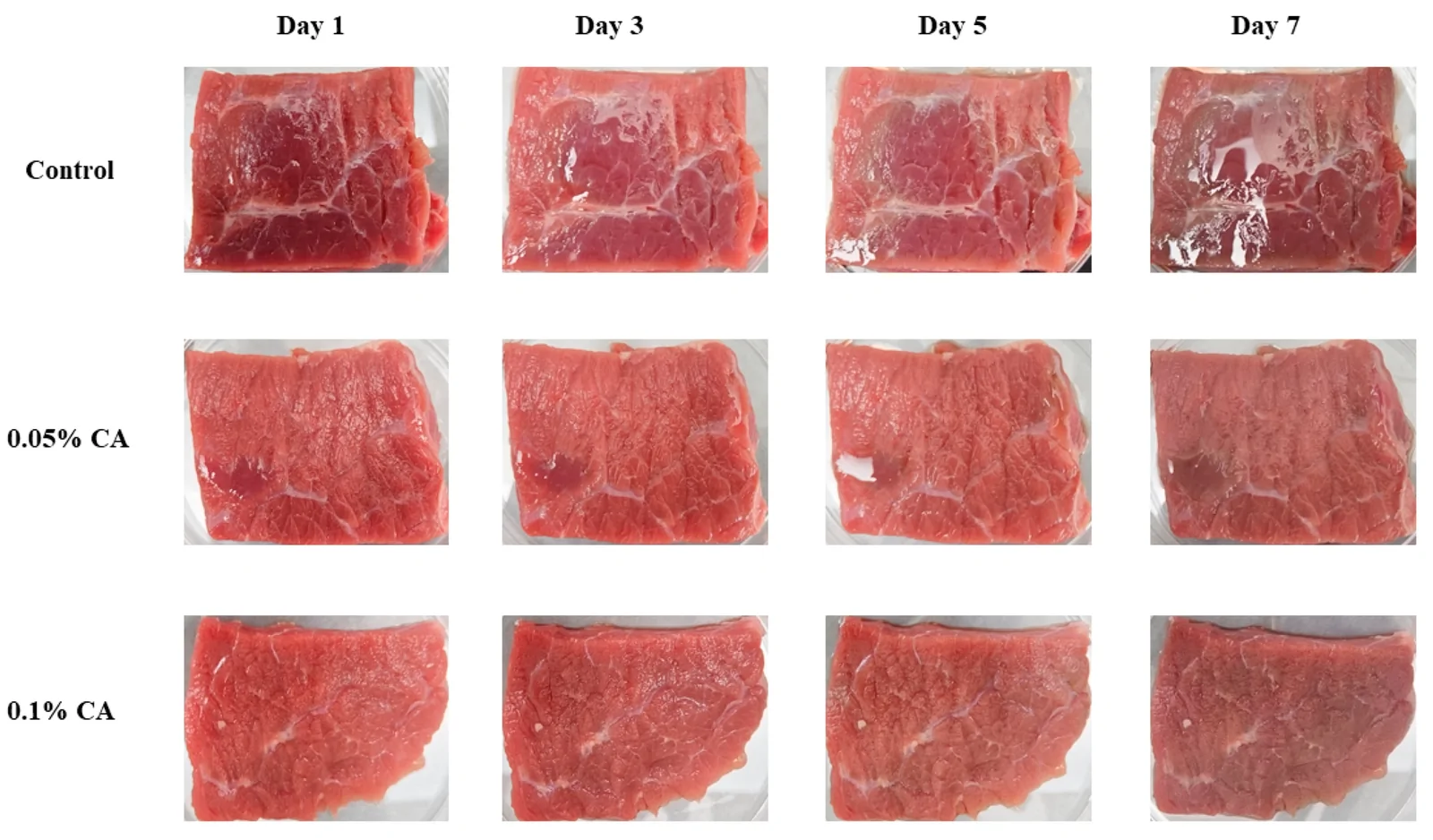
The effect of different concentrations of CA on the color of beef samples during storage.

**Figure 3:**
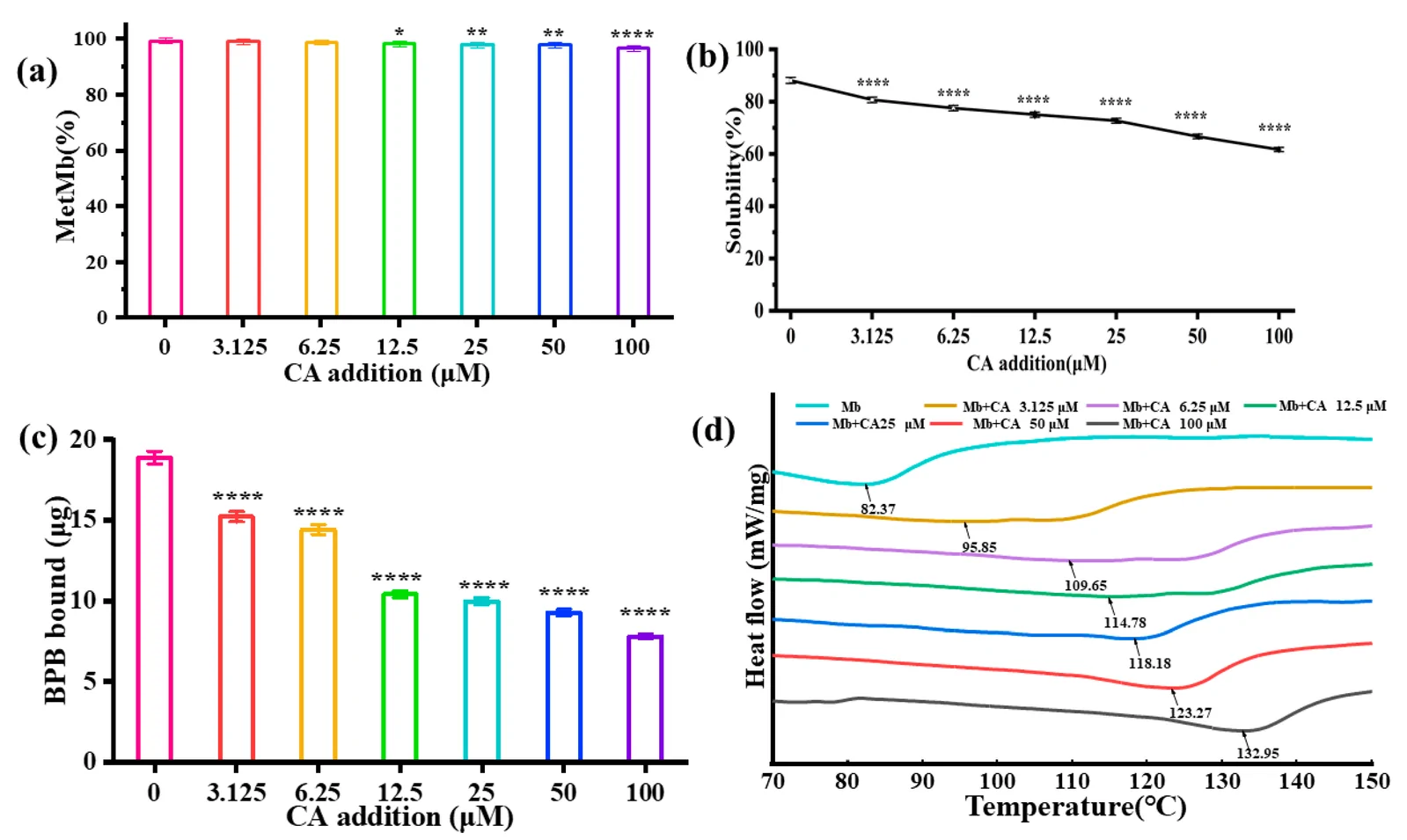
Effect of CA on MetMb% (**a**), solubility (**b**), surface hydrophobicity (**c**), and thermal stability (**d**) of Mb and Mb-CA complex (* *p* < 0.05, ** *p* < 0.005, *** *p* < 0.001, **** *p* < 0.0001 vs. no CA group).

**Figure 4:**
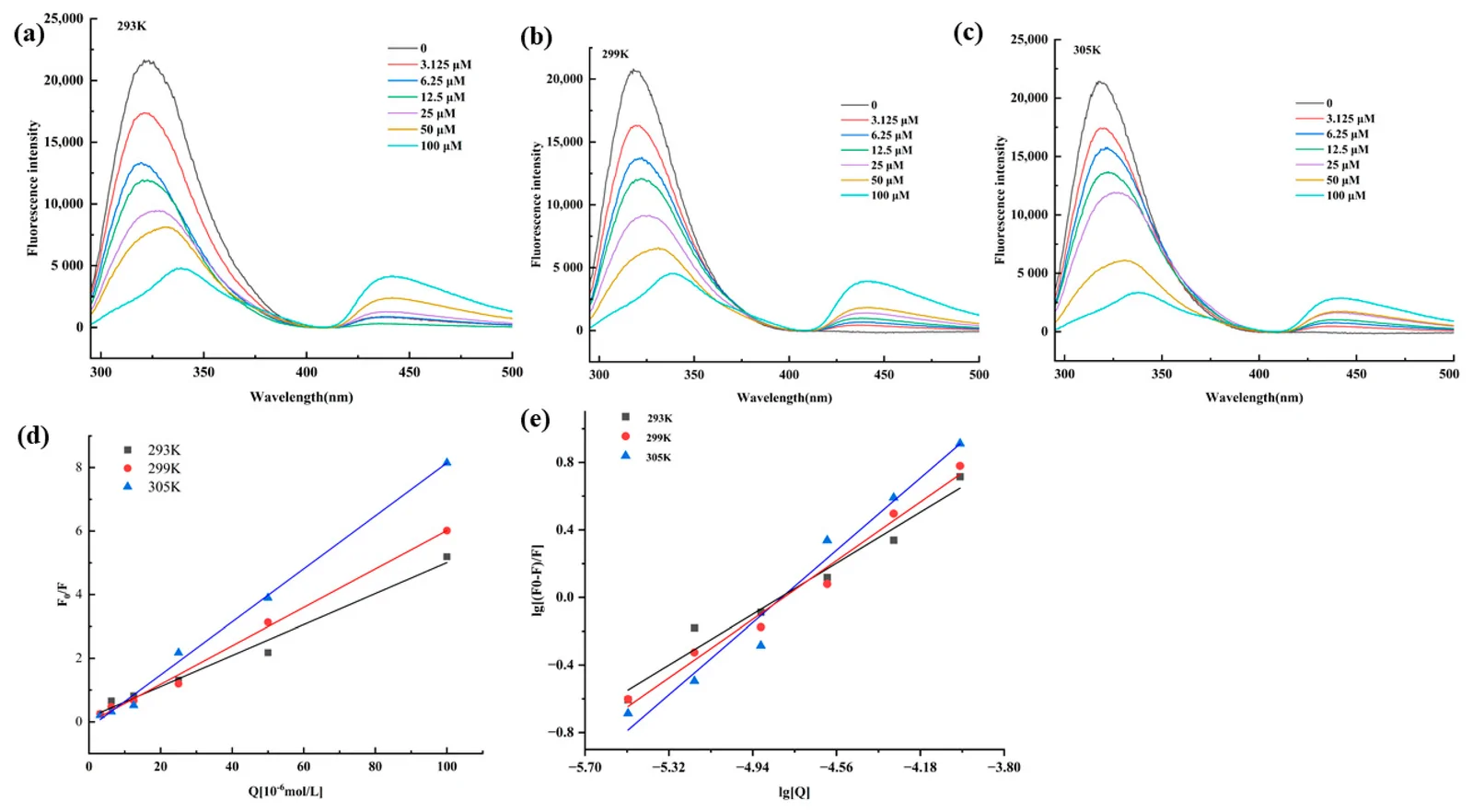
Fluorescence spectra of Mb treated with CA at 293 K (**a**), 299 K (**b**), and 305 K (**c**). Stern–Volmer plot of interaction between Mb and CA at three temperatures (**d**). The double logarithmic equation plot of Mb and CA (**e**).

**Figure 5:**
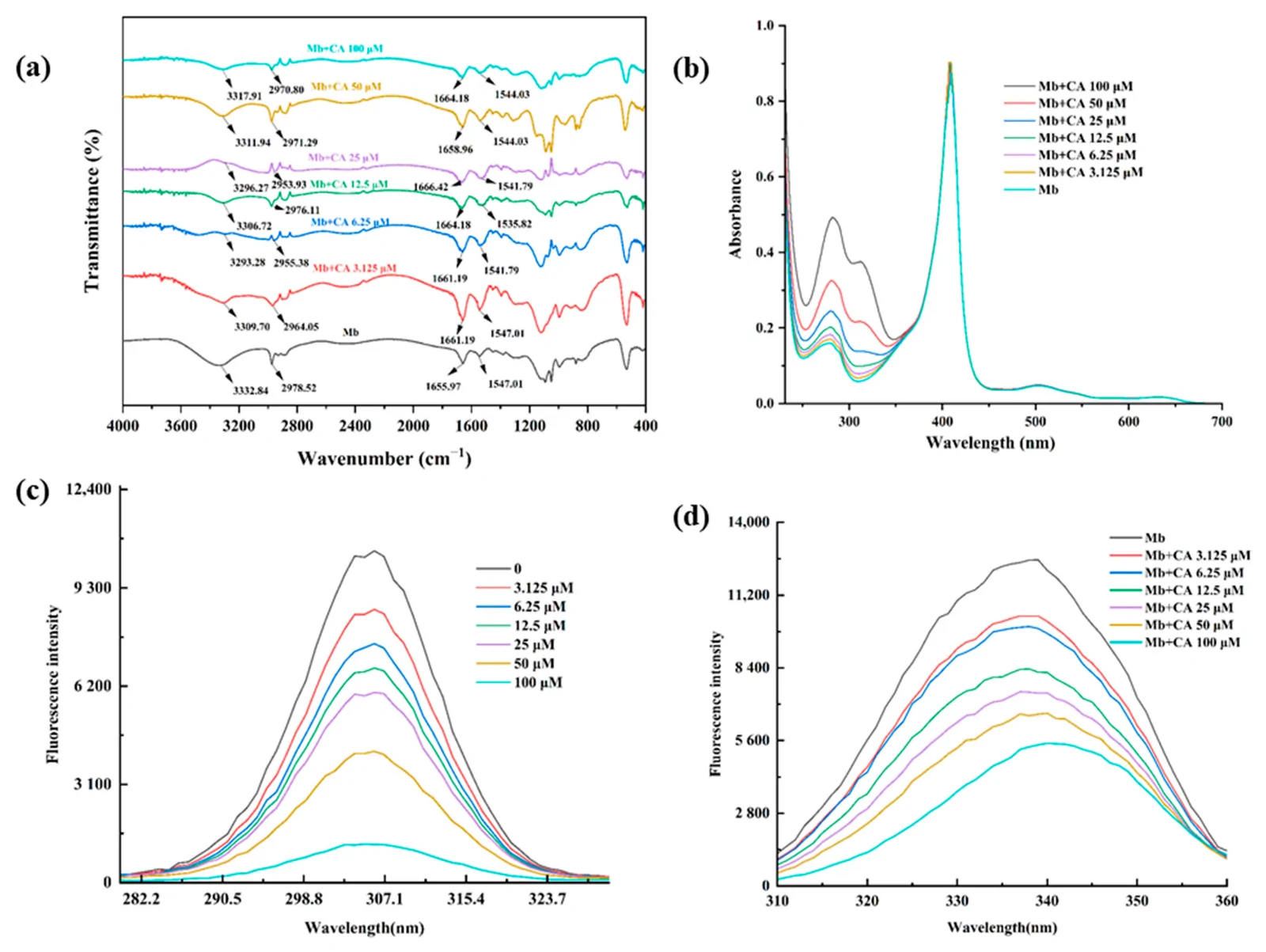
FT-IR spectra (**a**) and UV- visible spectra (**b**) of Mb and Mb-CA complexes. The synchronous fluorescence spectrum of Mb in the absence and presence of CA at Δλ = 15 nm (**c**) and Δλ = 60 nm (**d**).

**Figure 6:**
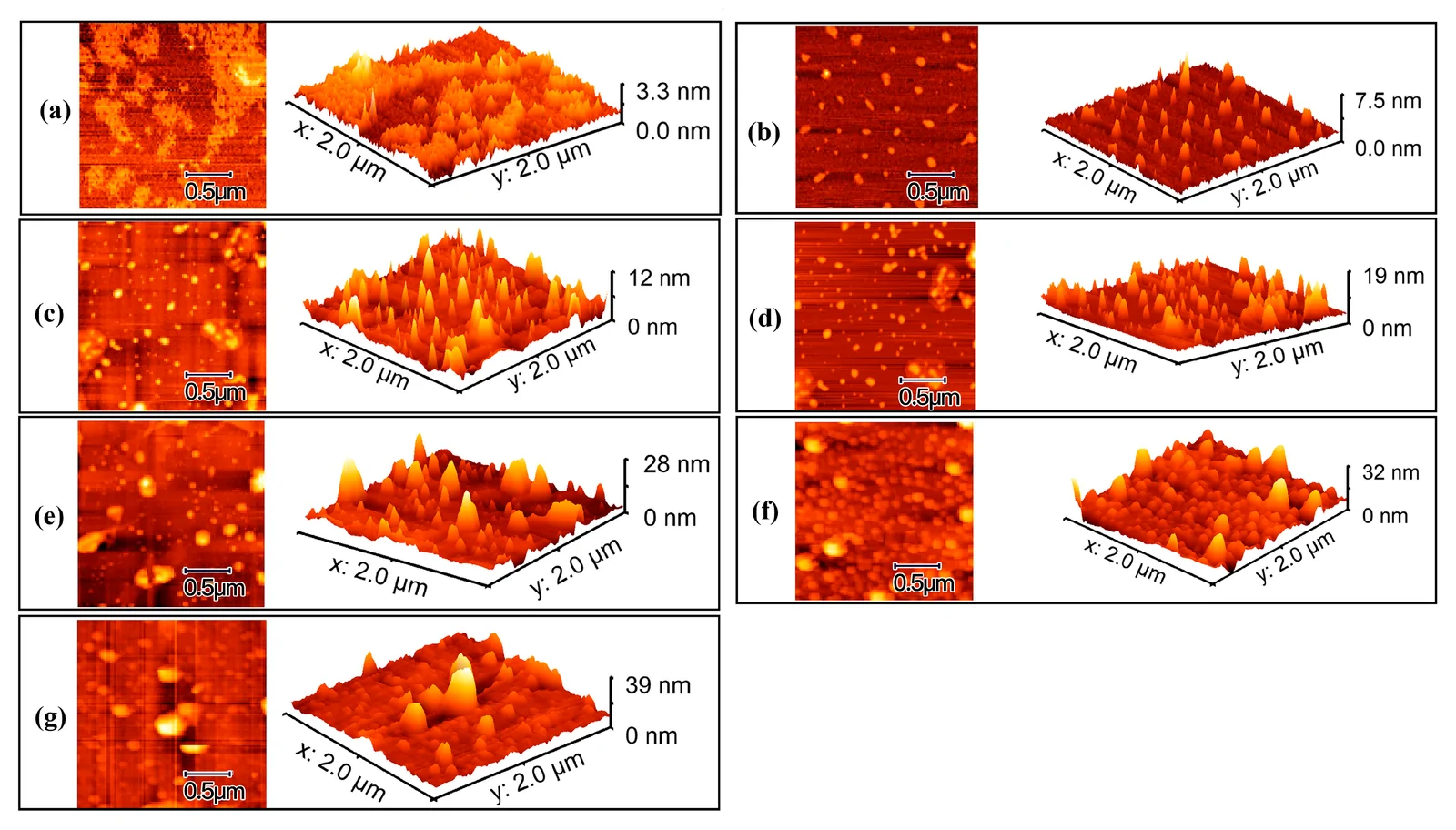
2D and 3D AFM images of Mb and Mb-CA complexes: 0 μM CA (**a**), 3.125 μM CA (**b**), 6.25 μM CA (**c**), 12.5 μM CA (**d**), 25 μM CA (**e**), 50 μM CA (**f**), 100 μM CA (**g**).

**Figure 7:**
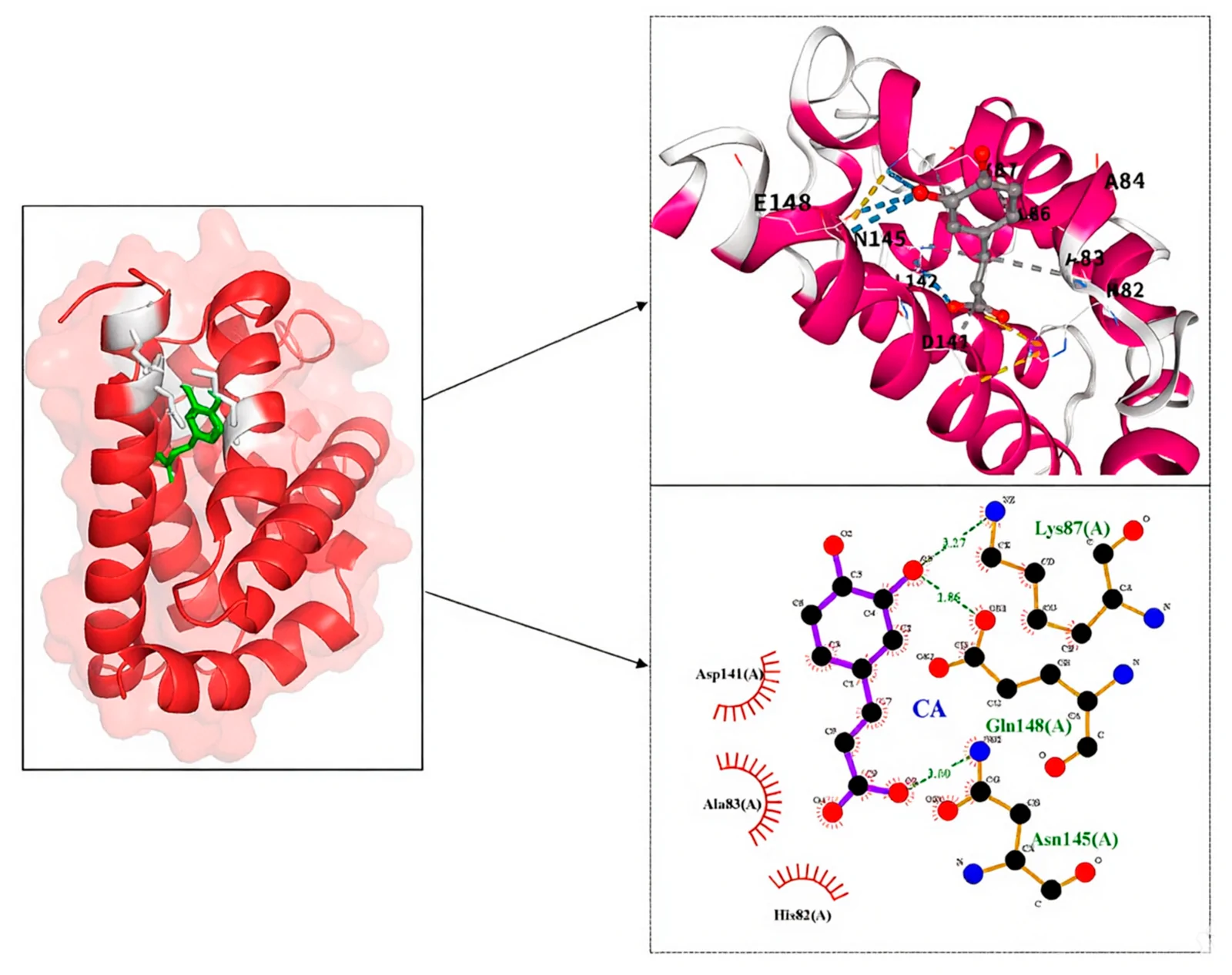
The molecular docking results of Mb with CA.

**Figure 8:**
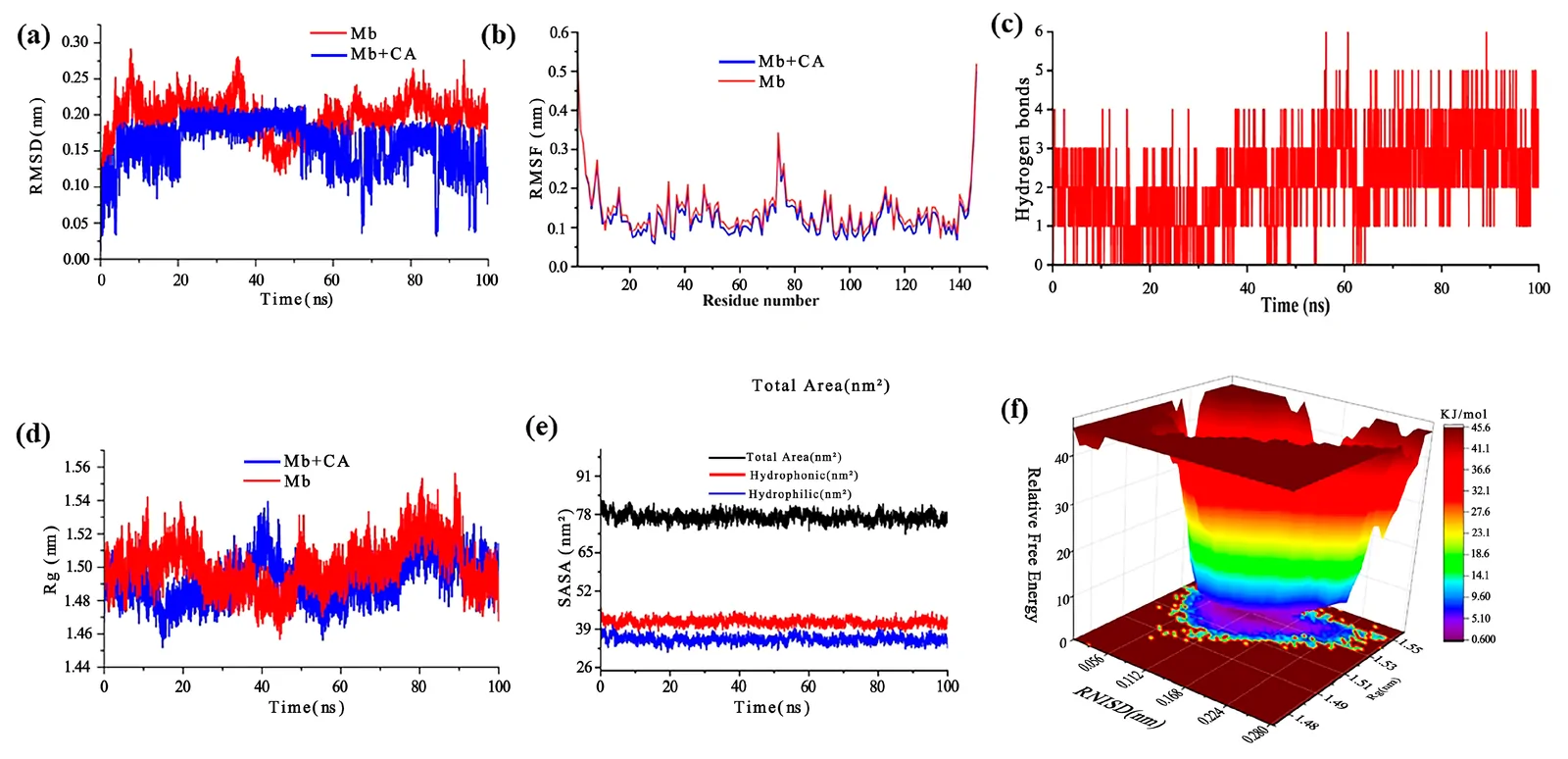
Molecular dynamics results of Mb and CA, RMSD (**a**), RMSF (**b**), number of hydrongen bonds (**c**), Rg (**d**), SASA (**e**), and FEL (**f**).

**Table 1:** Effects of different concentrations of CA on beef color.

Attribute	Display Time (Day)	Sample ID		
		Control	0.05% CA	0.1% CA
L*	1	44.92 ± 0.23 aA	44.39 ± 0.27 aAB	44.06 ± 0.27 aA
	3	45.46 ± 0.33 aA	45.40 ± 0.26 aB	44.52 ± 0.25 aA
	5	44.52 ± 0.32 aA	44.61 ± 0.32 aAB	44.38 ± 0.27 aA
	7	42.42 ± 0.43 aA	42.88 ± 0.57 aA	42.37 ± 0.42 aA
a*	1	15.28 ± 0.13 aC	15.80 ± 0.29 aBC	15.84 ± 0.19 aB
	3	13.79 ± 0.32 aB	16.06 ± 0.27 bC	16.78 ± 0.29 bC
	5	11.40 ± 0.44 aB	15.76 ± 0.20 bB	16.65 ± 0.21 bBC
	7	8.11 ± 0.16 aA	10.91 ± 0.22 bA	10.98 ± 0.24 bA
b*	1	9.49 ± 0.23 aB	9.72 ± 0.16 bAB	9.17 ± 0.080 aA
	3	9.24 ± 0.36 aA	9.84 ± 0.24 bAB	9.85 ± 0.20 bAB
	5	9.03 ± 0.38 aA	9.38 ± 0.23 bA	9.88 ± 0.19 bB
	7	8.32 ± 0.24 aA	9.13 ± 0.30 bB	9.64 ± 0.32 bB

Different lowercase letters on the same line indicate significant differences, and different uppercase letters on the same column in the same group indicate significant differences (*p* < 0.05).

**Table 2:** The quenching constants, bind constants and thermodynamic parameters for myoglobin-CA complexes at 293 K, 299 K and 305 K.

T (K)	K_SV_ (10^4^ L/mol)	Kq (10^12^ L/mol·s)	Ra^2^	Ka (10^3^ L/mol)	n	R_b_^2^	ΔG (kJ/mol)	ΔH (kJ/mol)	ΔS (kJ/mol⋅K)
293	8.33 ± 0.76 a	8.33 ± 0.76 a	0.989 ± 0.02	259.41 ± 0.23 a	1.13 ± 0.03 a	0.989 ± 0.03	−22.18 ± 2.26 b	−227.14 ± 5.12	−0.672 ± 0.04
299	6.04 ± 0.34 b	6.04 ± 0.34 b	0.997 ± 0.01	24.13 ± 0.45 b	0.91 ± 0.01 b	0.979 ± 0.01	−26.21 ± 2.13 a		
305	4.87 ± 0.12 c	4.87 ± 0.12 c	0.996 ± 0.03	6.66 ± 0.21 c	0.79 ± 0.02 c	0.987 ± 0.02	−30.24 ± 1.21 a		

Different letters in the same column indicate significant differences (*p* < 0.05).

## Data Availability

The original contributions presented in the study are included in the article; further inquiries can be directed to the corresponding author.

## Abbreviations

The following abbreviations are used in this manuscript:

CA	Caffeic acid
Mb	Myoglobin
MetMb	Metmyoglobin
DSC	Differential scanning calorimetry
AFM	Atomic Force Microscope
FT-IR	Fourier transform infrared
UV	Ultraviolet–visible
MD	Molecular dynamics simulation
RMSD	root-mean-square deviation
RMSF	root-mean-square fluctuation
Rg	radius of gyration
SASA	solvent-accessible surface area
FEL	Free energy landscape
